# Ancient eudicot hexaploidy meets ancestral eurosid gene order

**DOI:** 10.1186/1471-2164-14-S7-S3

**Published:** 2013-11-05

**Authors:** Chunfang Zheng, Eric Chen, Victor A Albert, Eric Lyons, David Sankoff

**Affiliations:** 1 Department of Mathematics and Statistics, University of Ottawa, 585 King Edward Avenue, Ottawa, Canada K1N 6N5; 2 Department of Biology, University of Ottawa, 30 Marie-Curie, Ottawa, Canada K1N 6N5; 3 Department of Biological Sciences, University at Buffalo, Buffalo, NY 14260, USA; 4 School of Plant Sciences, University of Arizona, 1140 E. South Campus Drive, Tucson, AZ 85721, USA

## Abstract

**Background:**

A hexaploidization event over 125 Mya underlies the evolutionary lineage of the majority of flowering plants, including very many species of agricultural importance. Half of these belong to the rosid subgrouping, containing severals whose genome sequences have been published. Although most duplicate and triplicate genes have been lost in all descendants, clear traces of the original chromosome triples can be discerned, their internal contiguity highly conserved in some genomes and very fragmented in others. To understand the particular evolutionary patterns of plant genomes, there is a need to systematically survey the fate of the subgenomes of polyploids, including the retention of a small proportion of the duplicate and triplicate genes and the reconstruction of putative ancestral intermediates between the original hexaploid and modern species, in this case the ancestor of the eurosid clade.

**Results:**

We quantitatively trace the fate of gene triples originating in the hexaploidy across seven core eudicot flowering plants, and fit this to a two-stage model, pre- and post-radiation. We also measure the simultaneous dynamics of duplicate orthologous gene loss in three rosids, as influenced by biological functional class. We propose a new protocol for reconstructing ancestral gene order using only gene adjacency data from pairwise genomic analyses, based on repeating MAXIMUM WEIGHT MATCHING at two levels of resolution, an approach designed to transcend limitations on reconstructed contig size, while still avoiding the ambiguities of a multiplicity of solutions. Applied to three high-quality rosid genomes without subsequent polyploidy events, our automated procedure reconstructs the ancestor of the eurosid clade.

**Conclusions:**

The gene loss analysis and the ancestor reconstruction present complementary assessments of post-hexaploidization evolution, the first at the level of individual gene families within and across sister genomes and the second at the chromosome level. Despite the loss of more than 95% of gene duplicates and triplicates, and despite major structural rearrangement, our reconstructed eurosid ancestor clearly identifies the three regions corresponding to each of the seven original chromosomes of the earlier pre-hexaploid ancestor. Functional analysis confirmed trends reported for more recent plant polyploidy events: genes involved with regulation and responses were retained in multiple copies, while genes involved with metabolic processes were lost.

## Introduction

The publication of the grapevine genome sequence by Jaillon *et al. *in 2007 [1] included the discovery of an ancient hexaploidization event, which also showed clear traces in all the other eudicot genomes sequenced up to that time, but which was absent from the monocot genome of rice. Since then, this event has been confirmed [2] and characterized in most detail with the publication of the cacao genome sequence [3] and in other work of Salse and colleagues [4]. It also has been dated to occur before the radiation of the core eudicots, but after their divergence from more basal eudicots [5]. The original basis for all this work was the observation of seven triples of homeologous regions in each genome. The ancient hexaploid would have combined three identical or closely related subgenomes, each made up of seven chromosomes. In the modern species, each of the three regions (fragmented by post-hexaploid genome rearrangements in some of the genomes), reflecting the three equivalent chromosomes in the original subgenomes, contains some genes paralogous to genes in one, or occasionally both, of the other two homeologous regions. Meticulous work identified the boundaries of these regions and suggested a credible history of chromosome fusions and other major rearrangements leading to the modern genomes from an ancestral *N *= 3 × 7 = 21-chromosome hexaploid.

There is a growing literature on gene order reconstruction in the context of flowering plant phylogeny [6-8], although the extensive paralogy induced by hexaploidization, and the subsequent gene order scrambling due to fractionation, i.e., the loss of duplicate genes from one or two of the three homeologous regions, more or less randomly, cause difficulty for the inference of ancestral gene order.

In this paper we follow two separate lines of investigation, the first into the dynamics of fractionation in seven descendants of the hexaploidization and functional constraints on which genes have lost copies, and the second into the reconstruction of an early descendant of the hexaploid, namely the common ancestor of the eurosids. Merging the two approaches combine both an intensional measure - detailed rates of gene loss - and an extensional characterization - changes to chromosomal structure - of the early evolution of the core eudicots.

**Figure 1:**
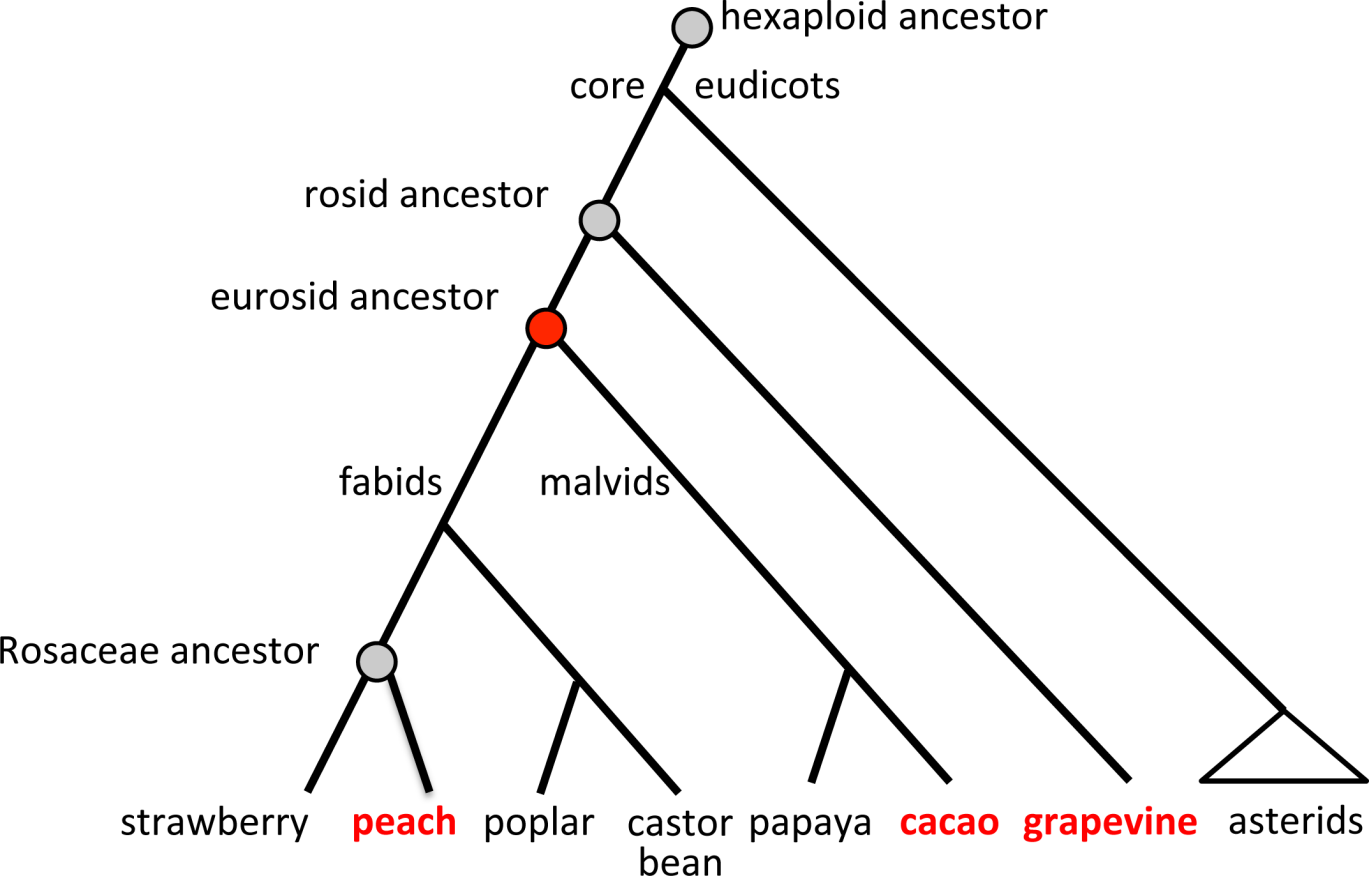
**Lineages of the rosids within the core eudicots**. Eurosid ancestor to be reconstructed on the basis of grapevine, cacao and peach, all in red. Only poplar among the genomes shown has had a recent whole genome duplication. All of the published asterid genome sequences and almost all other sequenced rosids not included in this study have undergone one or more polyploidy events since the core eudicot radiation.

The seven rosid genomes we analyze include six that have experienced no further polyploidization since their common origin (~110 Mya): *Vitis vinifera *(grapevine) [1,9], *Carica papaya *(papaya) [10], *Ricinus communis *(castor bean) [11], *Theobroma cacao *(cacao) [3] and *Fragaria vesca *(strawberry) [12], *Prunus persica *(peach) [8] as well as one that has undergone a tetraploidization (~70 Mya) since its lineage diverged from the others: *Populus trichocarpa *(poplar) [13]. Figure 1 summarizes the phylogenetic relationships among these species in the context of the core-eudicot clade. We also label four ancestral genomes, the hypothetical 21-chromosome hexaploid ancestor, the rosid ancestor, the eurosid ancestor and the 9-chromosome Rosaceae ancestor reconstructed in [8] and, schematically, in [4].

For the reconstruction of the eurosid ancestor, we use the grapevine genome, member of the earliest branching rosid order, Vitales, as an outgroup, as well as the two least rearranged eurosid genomes, cacao and peach. Our entirely automated method for the reconstruction of ancestral gene order

• starts from complete sets of orthologs inferred for pairs of genomes by SYNMAP in the COGE platform [14-16],

• harmonizes them into triples of orthologs across the three genomes by the OMG! technique [17],

• optimally infers ancestral contigs using MAXIMUM WEIGHT MATCHING (MWM) [18,19] on oriented (same or different DNA strand) gene adjacencies, and then discards any matches not supported by at least two of the genomes,

• replaces all the genes on the genomes by oriented symbols representing the new contigs,

• does another round of MWM on the adjacencies of the new symbols, to form chromosomal fragments,

• merges adjacent fragments if they are not separated by more than a preset number of genes on at least two genomes.

In contrast to genome halving [20] or genome aliquoting [21] methods, we do not attempt to reconstruct the ancestor at the moment of polyploidization. Rather we reconstruct a presumably rediploidized, partially fractionated, and somewhat rearranged descendant of that polyploid, in an effort to infer recent common ancestor of the extant genomes.

Before this reconstruction of the ancestral genome, which makes no reference whatsoever to hexaploidy or its remnants, we quantitatively analyze the internal paralogies, or homeologies, of the seven genomes, documenting the pervasive pattern of triples of syntenic blocks that are the signature of hexaploidization. We also develop a model for the fractionation process to account for the proportion of gene triples, gene pairs, and single copy genes within each genome. We attribute some of the deviation between model predictions and observations to rate dependence on gene functional class, and undertake to quantify this rate diversity based on known gene ontology in *Arabidopsis *of triplets, duplicates and single-copy of homologous rosid genes.

In the final sections of this paper, we apply the knowledge we have generated about the remnants, in the grapevine, cacao and peach genomes, of the 21 regions produced by the hexaploidization, to try to identify these regions in the ancestral eurosid genome we reconstructed, and find striking coherence between the reconstructed chromosomes and what would be expected from a gross comparison of the genomes at the level of entire regions.

## Gene triplicates resulting from the hexaploidization event

**Figure 2:**
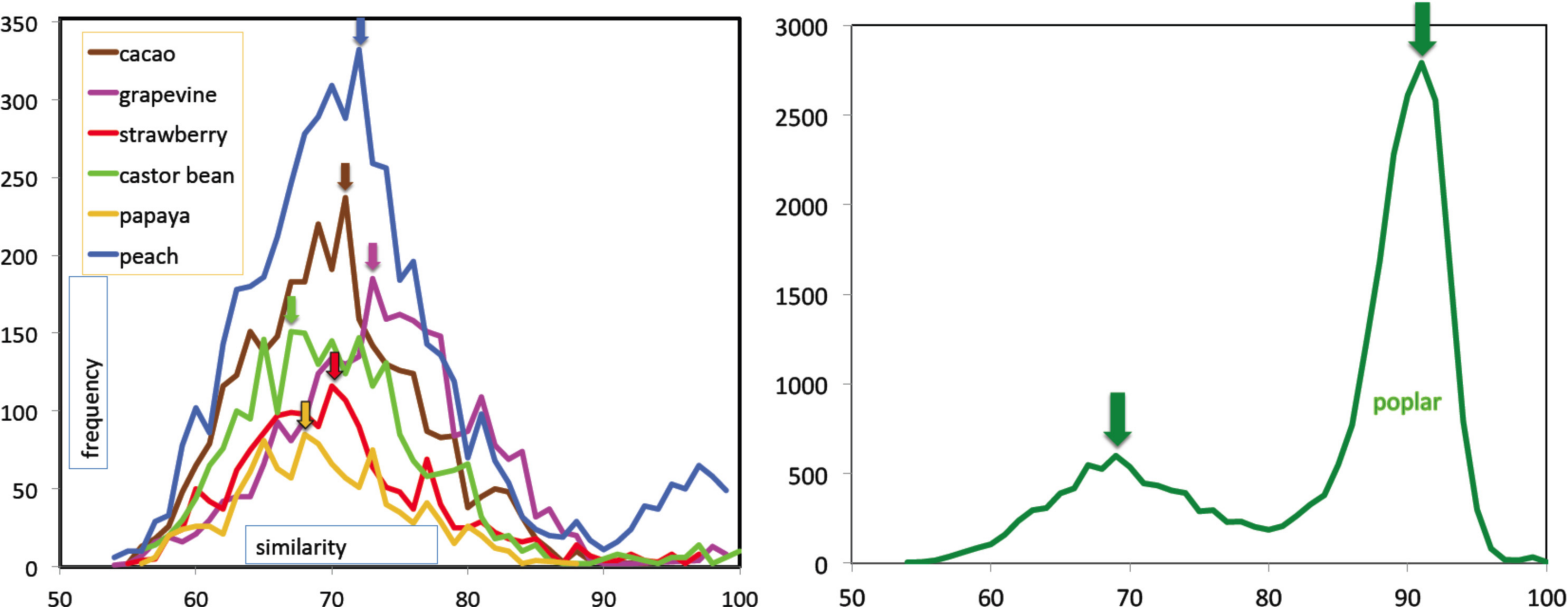
**Distribution of similarities in gene pairs**. Duplicate genes in seven genomes, showing triplication peaks between 67% and 73%. Poplar WGD peak at 91%.

Using SYNMAP to locate all synteny blocks of minimum size 5 in a self-comparison of each of the seven genomes revealed thousands of syntenic gene pairs, whose sequence similarities are plotted in Figure 2. Experience has shown (as reflected in the default values within SYNMAP) that the threshold of 5 genes anchoring a syntenic block is as low as we can set without admitting large amounts of noise into genomic comparisons. All of the genomes show a clear peak at 70% *± *3% sequence similarity. In addition, poplar showed a larger peak at 91%, reflecting its more recent WGD.

Syntenic dot-plots for the self-comparison of both grapevine and of cacao clearly show a pattern of pairwise homologies falling largely into 21 groups according to the chromosomal location of the two homologs [1,3]. This is also true to a lesser extent for peach. Within each of the three genomes, these groups of homologies are distributed among seven triples of large regions, where there are numerous homologies between each of the three *pairs *of regions. There are very few, if any, gene homologies within regions, few between different (non-homeologous) tripled regions, and few between genes in regions and genes outside of all tripled regions.

We adopt the coloring scheme introduced in [1,3] for the seven triples of regions: red, orange, yellow, green, pale blue, blue and purple. We distinguish between the three homologs of each colour by using dark, medium and light shades of each, ordered according to the number of genes detected in each by our methods, with the darkest containing the most genes.

For grapevine, cacao and peach, the ranges of the triplicated regions were determined by examining the dot plots produced by SYNMAP. For the other four genomes, the greater degree of chromosomal rearrangement entails more than 21 groups of smaller syntenic regions in the SYNMAP output. But these regions can still be grouped into triples, with numerous homologies between each of the three pairs of groups of regions and few homologies within groups, between genes in different triples, or between genes in groups and those in no group. Without risk of ambiguity we will refer to these groups of regions as "regions" as if they were each contiguous regions as in grapevine, cacao and peach.

**Figure 3:**
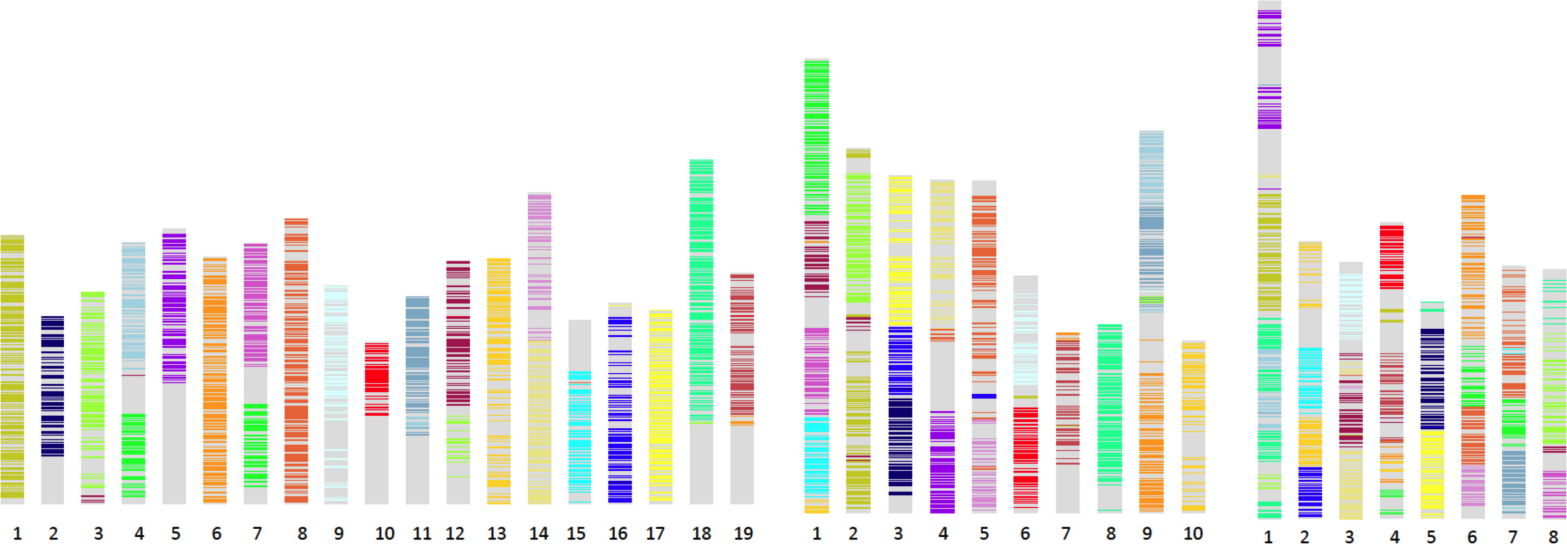
**Triplication in three rosid genomes**. Chromosomal distribution of triplicated regions in the grapevine, cacao and peach genomes. Colour intensities represent regions with more (darkest) or fewer (lightest) retained genes. Grey areas represent genes that are not in coloured regions.

Figure 3 depicts the distribution of the 21 regions created by the hexaploidization event in the 19 chromosomes of grapevine, the 10 chromosomes of cacao and the 8 chromosomes of peach. These regions are slightly extended in each genome by adding genes that are not necessarily adjacent to the original regions, but are identified through their homologies with coloured regions in the other two genomes.

Due to rapid fractionation, there are very few *triples *of homologous genes within each genome, as we will discuss in the next section. This loss of iso-functional paralogs results in an *interleaving *[22] pattern of pairwise homologies among three regions.

As we can see in Table 1, most of the duplicate genes in synteny blocks (produced by SYNMAP) are coloured. For a very large majority of the coloured blocks, there is only one colour. Importantly, all of the gene pairs with two coloured genes involve two separate regions (different shades in Figure 3) of the same colour. These observations can only be explained by a hexaploidization event being the origin of most of the gene pairs within these eudicot genomes.

**Table 1:** Distribution of colours among synteny blocks.

	genome						
	**peach**	**cacao**	**grapevine**	**castor bean**	**strawberry**	**papaya**	**poplar**

genes in synteny blocks	4326	2878	2435	2147	1576	1098	5202
% genes coloured	83	90	88	90	82	90	86
% genes coloured in coloured blocks	87	91	88	92	84	91	88

number of blocks (most counted 2X)	481	362	247	267	225	160	1155
% one colour	79	96	97	93	85	94	74
% no colour	12	1	1	2	4	1	19
% different colour	9	2	2	5	10	4	7

number of gene pairs (most counted 2X)	5121	3143	2709	2347	1638	1110	7273
% in coloured blocks	93	99	100	98	97	98	98
% same colour, different region	74	84	77	83	71	81	75
% same colour, same region	1	0	0	0	1	1	5
% one gene coloured	9	11	21	12	15	12	12
% neither coloured	7	1	0	2	3	2	2

Grapevine, cacao and peach coloured independently; the ranges of the coloured regions determined by inspection of the self-comparison dot-plots; for the first two, these correspond as closely as can be determined to the ranges displayed in [1] and [3]. The other four genomes are coloured according to homologies with the first three. Peach ranges are more difficult to distinguish than those of grapevine and cacao, accounting for the somewhat elevated number of blocks of different colors, and this ambiguity is projected via the coloring process onto strawberry and poplar.

## Fractionation of duplicates and triplicates

The number of genes in the common ancestor of the seven genomes is unknown; the suggested 10,000 genes in the pre-hexaploidy genome [3] (30,000 in the hexaploid) is likely a severe underestimate; plant genomes generally have more than 20,000. Between hexaploidization (time *t*_0_) and radiation of the core eudicots (time *t*_1_), many duplicates would have been lost. After radiation, loss would have continued *independently *in each lineage. Table 2 reports the number of triplets of homologous genes remaining today.

**Table 2:** Gene family sizes.

	frequencies of gene family sizes						
**Size**	**peach**	**cacao**	**grapevine**	**castor bean**	**strawberry**	**papaya**	**poplar**

2	1484	1111	945	851	606	474	1278 (3-4)
3	256	172	150	119	57	34	177 (5-6)
*≥ *4	21	5	12	2	9	1	16 (*>*6)

Numbers of triples and pairs after fractionation

Let *p *represent the probability that a redundant (duplicate or triplicate) gene would be lost during the time span from *t*_0 _to *t*_1_, from hexaploidization to the radiation, had there been no functional constraints. However, we can assume that the event where all three copies were lost was prohibited. Adapting a derivation for a different scenario of compound polyploidization [23], the probability that

1. all three genes survived is $\frac{{\left(1-p\right)}^{3}}{1-p^{3}}$.

2. two of the three survived is $\frac{3p{\left(1-p\right)}^{2}}{1-p^{3}}$, and

3. only one survived is $\frac{3p^{2}\left(1-p\right)}{1-p^{3}}$.

Similarly, let *q_i _*represent the probability that a redundant (duplicate or triplicate) gene would be lost from species *i *during the time span from *t*_1 _to *t*_2_, from the radiation to the present, were there no functional constraints. Then the probability that

4. a triplet would still survive is $\frac{{\left(1-p\right)}^{3}}{1-p^{3}}\frac{{\left(1-q_{i}\right)}^{3}}{1-q_{i}^{3}}$.

5. an original triplet would manifest as a pair is $\frac{{\left(1-p\right)}^{3}}{1-p^{3}}\frac{3q_{i}{\left(1-q_{i}\right)}^{2}}{1-q_{i}^{3}}+\frac{3p{\left(1-p\right)}^{2}}{1-p^{3}}\frac{{\left(1-q_{i}\right)}^{2}}{1-q_{i}^{2}}$, and

6. an original triplet would be reduced to a single copy is $\frac{{\left(1-p\right)}^{3}}{1-p^{3}}\frac{3q_{i}^{2}\left(1-q_{i}\right)}{1-q_{i}^{3}}+\frac{3p{\left(1-p\right)}^{2}}{1-p^{3}}\frac{2q_{i}\left(1-q_{i}\right)}{1-q_{i}^{2}}+\frac{3p^{2}\left(1-p\right)}{1-p^{3}}$.

**Figure 4:**
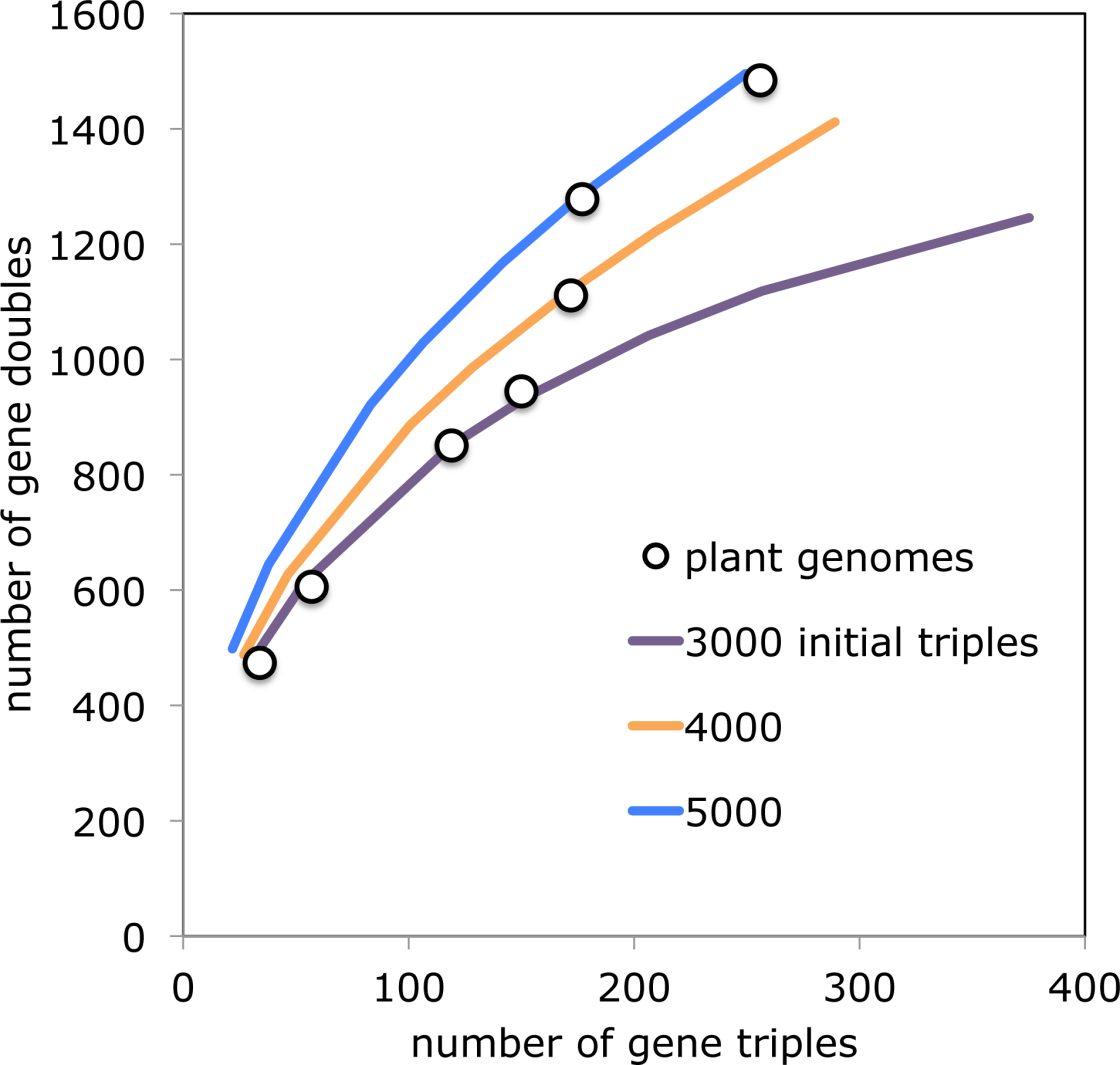
**Model and data**. Curves of duplicate-triplicate configurations in model, compared to values for seven rosids.

Using equations 4 and 5 to predict the numbers of triplicates and duplicates, assuming various numbers of original triplets, and maximum likelihood estimates of *p *and lineage-specific *qi*'s, produces the curves in Figure 4. When the data for the eudicot genomes are plotted, it is clear they behave as if the original number of triplets was much less than 10,000. A partial explanation is that a single parameter *p *and a single *q_i _*for each lineage is too simple a model. Not only can fractionation proceed at different rates in different lineages, its pace can also differ for different classes of genes [24,25]. (Cf. [26] for a study of rate inhomogeneities in an amphibian tetraploid.)

Though a general functional classification of all or most genes is not available for any of our rosid genomes, in the next section we initiate a comparative study of fractionation rates by assuming that genes have the same functions as their *Arabidopsis *homologs, when this homology can be detected.

## Functional analysis

We carried out a search for *Arabidopsis *orthologs for the genes in our grapevine, cacao and peach data by subjected them to the LastZ analysis in CoGe against the TAIR Version 10 database at http://arabidopsis.org/. For each rosid gene we picked the best *Arabidopsis *hit. Only the three genomes were examined because for comparability, our subsequent analysis required at least one copy of a gene in each genome, and no more than three. We found a total of 7200 such *complete *gene families in the three genomes; including the remaining genomes in the analysis would have drastically diminished this number and compromised the statistics.

Gene ontologies for all the *Arabidopsis *homologs chosen were downloaded from http://www.geneontology.org/GO.downloads.ontology.shtml. For 84% of the 7200 gene families, all the genes mapping to an *Arabidopsis *homolog mapped to the same one. For those families containing members mapping to different homologs, these were virtually always *Arabidopsis *genes having the same functionality.

For the 7200 gene families, we distinguished five categories of multiple-copy gene retention: 1 = gene families consisting of only one copy in all three genomes, 2 = genes that have one copy in two of the genomes and two or three copies in the third genome, 3 = genes that have one copy in one genome and two or three copies in the others, 4 = genes with two copies in each genome, 5 = gene families with at least two copies in each genome, at least one of which contains three copies. Thus genes with lower scores have lost more copies to fractionation and those with higher scores have retained more and are "fractionation-resistant".

For each family, we tabulated all the GO terms "hit" by at least one member, and amalgamated these terms for the family. We noted that, not surprisingly, families in higher scoring categories, containing more genes, hit a higher number of terms than families with lower scores. To see if some gene functions were associated with higher or lower rate of retention, we normalized the proportions of gene families in each retention category showing an association with a term by the proportion of gene families in that retention category showing *any *hits, separately for *Biological function, Cellular component *and *Molecular function*. For example, 3982/5933 = 67% of families in retention category 1 (one copy in each genome) had at least one hit for some *Biological function *term. One of these terms, *Response to stimulus *was hit by 15% of these families, so that the normalized association value for this GO term and this retention category is 0.15/0.67 = 0.22.

**Figure 5:**
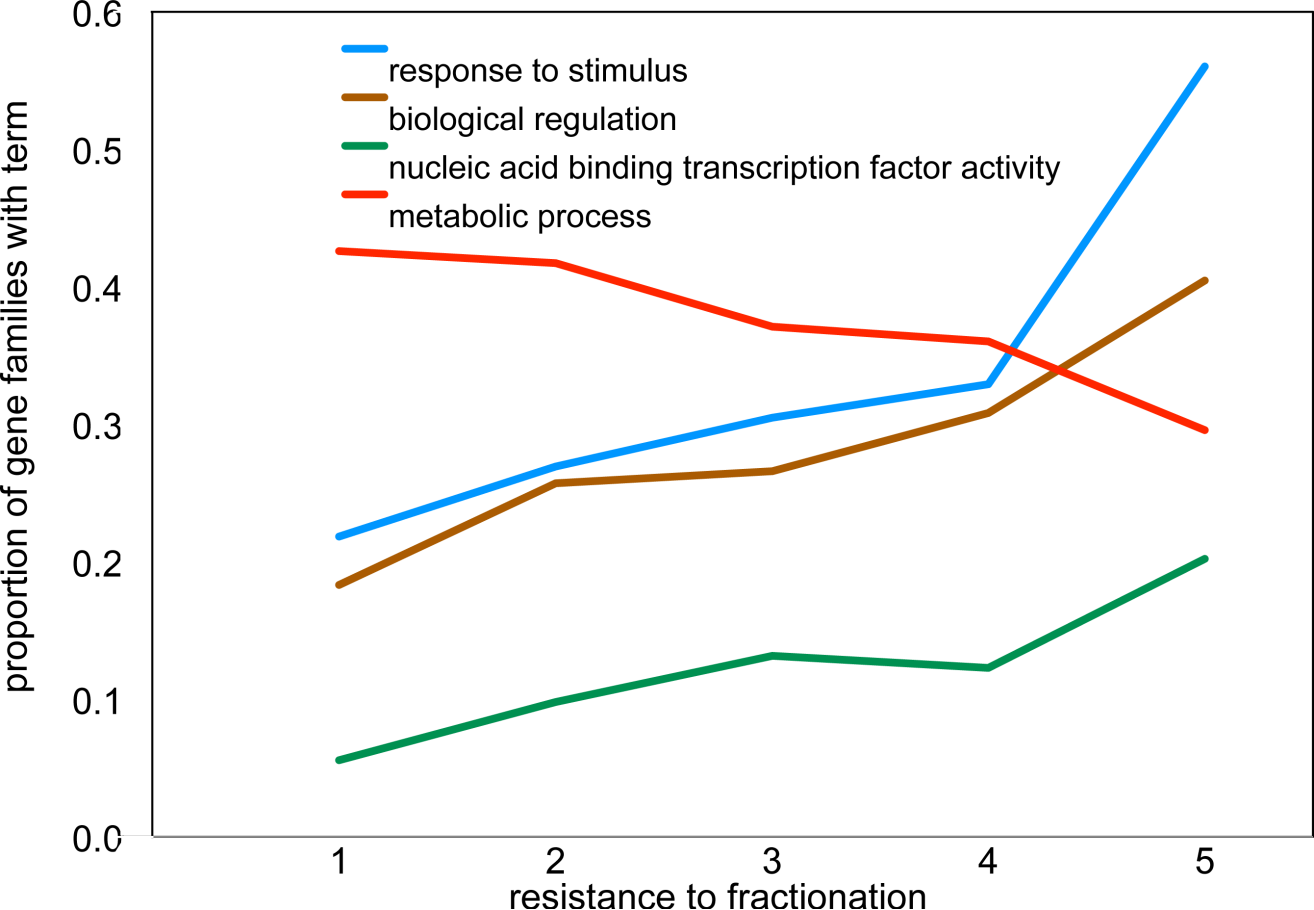
**Fractionation and function**. Functional categories of fractionation-resistant and non-resistant gene triplicates.

Figure 5 shows, out of 67 general functional categories, four with a significant number of hits exhibited a clear trend, either increasing with fractionation retention (or *resistance*) (four of them) or decreasing (one). The association of retention with regulatory and response terms has been shown in connection with more recent polyploidization events [24,25], as is the tendency for loss of duplicate genes in some metabolic families. Of interest is that this latter decreasing trend is in contrast with the GO term for *Regulation of metabolic process*, which is clearly associated with high retention gene families.

## The reconstruction

### Matching

The formal structure we adopt, both for the extant rosid genomes used as input data, and for the ancestral eurosid we aim to reconstruct, is the *oriented gene order*. In graph theoretical terms, these are pairs of graph matchings. The set of vertices of the graphs contains the two ends of all the genes, distinguished either as 5' versus 3', *h*(ead) versus *t*(ail) or "+" versus "-". The first matching simply connects every gene end to its other end. (Because every vertex is connected to another vertex, this is a *complete *matching.) The second connects each gene end to at most one other gene end (usually of a different gene), representing the adjacency of two genes. This is usually not a complete matching; a vertex representing a gene end at the end of a chromosomes is not connected to any other vertex.

### The median problem

The problem of reconstructing an unknown gene order on the basis of three given gene orders from related genomes is called the *median problem*. There are many methods for solving this (e.g. [27-30]); they generally attempt to construct a match as similar as possible, optimizing some measure of similarity under various constraints, to the three given adjacency matchings. The methods are distinguished by fine distinctions among the simplified models of genome rearrangement invoked, assumptions about identical gene complement or lack of duplicate genes, insistence or not on linearity of chromosomes or the number of chromosomes in the solution, relative weights of adjacencies versus chromosome-terminal gene ends, and other considerations.

### Non-uniqueness and stepwise procedures

Most gene order median problems are difficult: exact algorithms bog down for realistic instances while heuristics risk somewhat sub-optimal solutions. But of much more concern to biologists than details about optimality and efficiency is that the reliance on global optimality criteria leads to a severe problem of non-uniqueness of solutions. Reconstruction methods based on gene-by-gene adjacencies do not scale well for gene orders involving tens of thousands of genes. Long reconstructed chromosomes inevitably contain many poorly supported adjacencies, i.e., adjacencies that only occur in one, or even none, of the three data genomes. Though these may be relatively infrequent in terms of overall proportions, for example, 1% of 1000 adjacencies, giving 10 per chromosome, this is still a worrisome number; the reconstructions can often be broken at these points and reassembled in different combinations using other hitherto unused adjacencies, without losing the optimality of the solution. As a result there are an exponential number of solutions, many of them differing greatly at the level of chromosome structure. This is a dismaying situation for a biologist.

This partially explains the pervasiveness, in the field of ancestral genome inference, of reconstructions in two or more steps. The first step reconstructs a large number of small "contigs" or anchors, and the subsequent steps pieces these together into larger chromosome fragments or whole chromosomes, using some other objective function or relying on some other data. Often the ultimate step in reconstruction involves manual intervention, invoking biological intuition or some implicit expectations about the final shape of the solution.

### Uniqueness as a priority

In this paper, we also use a multi-step process, but our goals are first, to eliminate as far as possible sources of non-uniqueness in the reconstruction. We make sure that nothing is reconstructed on the basis of a series of arbitrary choices among equally plausible alternatives (a recipe for rampant non-uniqueness), and second, that the whole process be automated and reproducible, with explicit thresholds and other parameters.

#### Step 1: Generating gene orders on sets of orthologous genes

The input to the reconstruction method is the complete gene order for each of the extant genomes, together with identification of orthologous genes in the different genomes. We construct our gene orders based on SYNMAP's pairwise comparisons of gene orders: grapevine-cacao, cacao-peach and peach-grapevine, using its rigorous default values to ensure that orthologs are identified not only by their sequence similarities, but also by severe requirements on their syntenic context (i.e., syntenic blocks are identified only if they contain at least 5 pairs of collinear orthologous genes). We impose transitivity on the results where this does not already obtain, e.g., if *a *is orthologous to *b *and *b *is orthologous to *c*, then *a *is assumed orthologous to *c *whether or not this has been detected by SYNMAP.

For the grapevine, cacao and peach genomes, this initially produced a total of 14,543 orthology sets containing two, three or more genes, for a total of 39,286 genes.

#### Step 2: Resolving paralogy

The requirement for synteny within SYNMAP is an effective way of resolving non-tandem paralogy in large majority of instances. Two paralogs in different syntenic contexts are treated separately as different genes by virtue of these contextual distinctions.

Paralogs still show up, however. Genes *a*_1 _and *a*_2 _in genome A and can both register as orthologous to gene *b *in genome B because of segmental duplication in A or because identical contexts of *a*_1 _and *a*_2 _survived the fractionation process intact since the early polyploidization process. *a*_1 _and *a*_2 _are thus paralogs.

We use the OMG! procedure [17] to judiciously suppress a small number of homology relations to resolve these situations, usually by dividing large orthology sets into smaller ones, allowing only three or two genes to be considered orthologous across the three genomes. The objective is to maximize the sum of the squares of the number of genes in remaining orthology sets (9 or 4, respectively, for three-way and two-way orthology).

For the grapevine, cacao and peach genomes, this produced a final total of 5664 orthology sets containing two genes and 9148 containing three genes, for a total of 14,812 sets containing 38,772 genes.

#### Step 3: Maximum Weight Matching

Because we will be weighting adjacencies differentially, we do not use any of the dedicated median solvers, but the more flexible approach of MAXIMUM WEIGHT MATCHING (MWM). This allows us to make a slight weight distinction between two conflicting matches that appear equally often in the three genomes, but in subtly different contexts. We solve the MWM problem on the three sets of adjacencies, one for each genome, determined by the order of the genes output by SYNMAP and OMG!. The first polynomial-time exact algorithm for MWM -- "path, trees and flower" -- was introduced by Edmonds [18]. We use Galil's *O*(*n*3) version of the algorithm [19] for which Python code by J. van Rantwijk is available online.

The weighting system we use is displayed in Table 3. The first subtlety we incorporate is that if a match is present in a certain number of genomes, the evidence for that match is stronger if one or both of the vertices are absent from the other genomes than if they are both in conflicting adjacencies in those genomes. The second subtlety is that if a match is present in one genome only, but it appears that it is absent from another only because there has been a chromosomal inversion disrupting it, then it should have a higher weight than other single-genome matches.

**Table 3:** Weight system for adjacencies.

occurrence of adjacency between gene ends *x *and *y*	weight of *xy*
*xy *occurs in all three genomes	3.00
*xy *occurs in two genomes; *x *or *y *is absent from the third genome	2.03
*xy *occurs in two genomes; *x *and *y *present in the third genome	2.00
*xy *and *uv *occur in one genome; *xu *and *yv *in another; *uv*, but not both *x *or *y*, in third	1.50
*xy *and *uv *occur in one genome; *xu *and *yv *in another; *x*, *y *and *uv *in third	1.49
*xy *occurs in one genome; one of *x *or *y *absent in each of the other genomes	1.01
*xy *occurs in one genome; *x *and *y *both present in one or neither of the other genomes	1.00
*xy *occurs in one genome; *x *and *y *present in other two genomes	0.99

Weight system for adjacencies

In the output from the algorithm, the 14,812 orthology sets were organized into 63 contigs. These included all the adjacencies in the input with weights 3.00 (5657 of them), 2.03 (6319), 2.00 (1109) or 1.50 (113), and all except one of weight 1.49 (31 of them). In addition there were 1520 of adjacencies of weight 1.01, 1.00 or 0.99.

#### Step 4: Eliminating sources of non-uniqueness

We know that it is the adjacencies of weight less than 2.00 that propagate non-uniqueness. Those with weight 2.00 or more are very likely to be in all solutions. In our particular data this extends to the adjacencies of weight 1.50 and almost all of weight 1.49.

If we discard all poorly supported adjacencies from the (MWM) solution, then, there can be little variability in the solutions. This, however, comes at a cost of increasing the number of contigs from 63 to 1583, which must then be assembled into chromosomes by other means.

One incidental benefit of discarding most low-weight adjacencies is that all the circular contigs among the 63 are broken into linear fragments. It is theoretically possible to have circular contigs containing only weight 2.00 adjacencies, but this is not encountered in our data.

In addition, we discard 1368 genes scattered among 934 short contigs (three adjacencies or less). The large majority of these have only one gene, and are likely to represent errors originating in SYNMAP or OMG!, or movements of very small genomic fragments, containing little information about the main evolutionary events in these genomes. They also represent possible sources of non-uniqueness in reconstruction of ancestral chromosomes.

Note that had we attempted to use any of the other sequenced rosids instead of, or in addition to grapevine, cacao and peach, few additional contigs could be expected, because the highly fragmented nature of the subgenomes in these genomes would have resulted in mostly short contigs that would only have been discarded; some existing contigs could have received additional support, but since all except the weight 1.00 adjacencies are already consistent among themselves, this would have been of little benefit.

#### Step 5: The summary genomes

We use the 649 remaining contigs to create "summary" versions of the original three genomes. Every contig in the solution to the MWM projects a coordinate position and an orientation back to a single chromosome in each of the grapevine, cacao and peach genomes (even though the contig may contain a minority of genes from another chromosome), based on the average coordinates and most frequent orientation of the genes it contains from that genome on that particular chromosome.

#### Step 6: MWM on summary genomes

We can thus recognize adjacencies between these contig projections in each summary genome and carry out MWM, using the same weights as in Table 3 in Step 3 and the deletions in Step 4 to construct a new median consisting of a matching on the contigs. Again we discard adjacencies supported by only one of the three genomes. There remain 177 new contigs.

#### Step 7: Merging contigs

We can then merge new contigs with neighboring projections in two or more genomes, according to a criterion of how many genes may intervene between two candidates for merger. For each pair of new contigs on the same chromosomes in at least two genomes, we do the following:

• decompose each contig into pieces that are internally strictly contiguous on the two genomes,

• find the two pieces, one from each new contig, that are the closest, when appropriately oriented.

We then merge the pair of new contigs that are the closest, as long as they are not more than 500 genes apart on either of the two genomes. The cutoff in this final step, 500 with our data, is critical, but is easy to find since it has a direct and dramatic effect on the result. Lower values, like 400, result in an unrealistically large number of small chromosomes; higher values like 600 produce assemblies including one or two impossibly long chromosomes.

#### Run times

The pipeline we have described is not computationally intensive. Data preparation Steps 1 and 2 required 1 to 2 minutes in total for the rosid data. The MWM run in Step 3 took 15 minutes. The data manipulations in Steps 4 and 5 required less than a minute. The second MWM executed in 2 seconds.

### Validation

**Figure 6:**
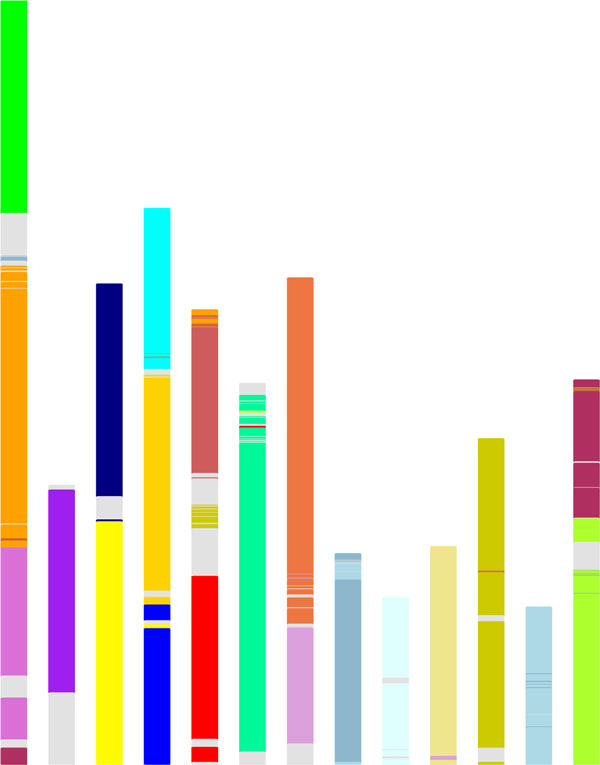
**Ancestral genome**. Distribution of triplicated regions in the eurosid ancestor.

The above procedure results a total of 13 chromosomes, as shown in Figure 6, where each gene is coloured by its triplication regions in grapevine, cacao and peach.

This shows that despite the fact that no triplication information whatsoever was used in the reconstruction, most of the 21 triplicated regions are located solely or largely on a single chromosome in the reconstruction. Moreover, as we shall see in the next section, the general structure of the chromosomes corresponds closely to what we would expect from common patterns of region fusions in the three genome.

## Reconstruction by shared fusions

Inspection of the coloured karyotypes in Figure 3 is suggestive of some of the evolutionary events which must have transpired in the rosids, without need to reference the individual genes that make up the triplicate regions, in the style of [3,4]. The main principles we adopt are first, that these genomes are descendants of a hexaploid, and second, that syntenic segments from two ancestral chromosomes on a single modern chromosome, when observed in two or three of the sequenced genomes, is unlikely to have been produced by two or three coincidental events, but is rather most parsimoniously accounted for by a single event at some point in the common evolutionary history of these genomes. This ties in with our search for uniqueness of reconstructions.

**Figure 7:**
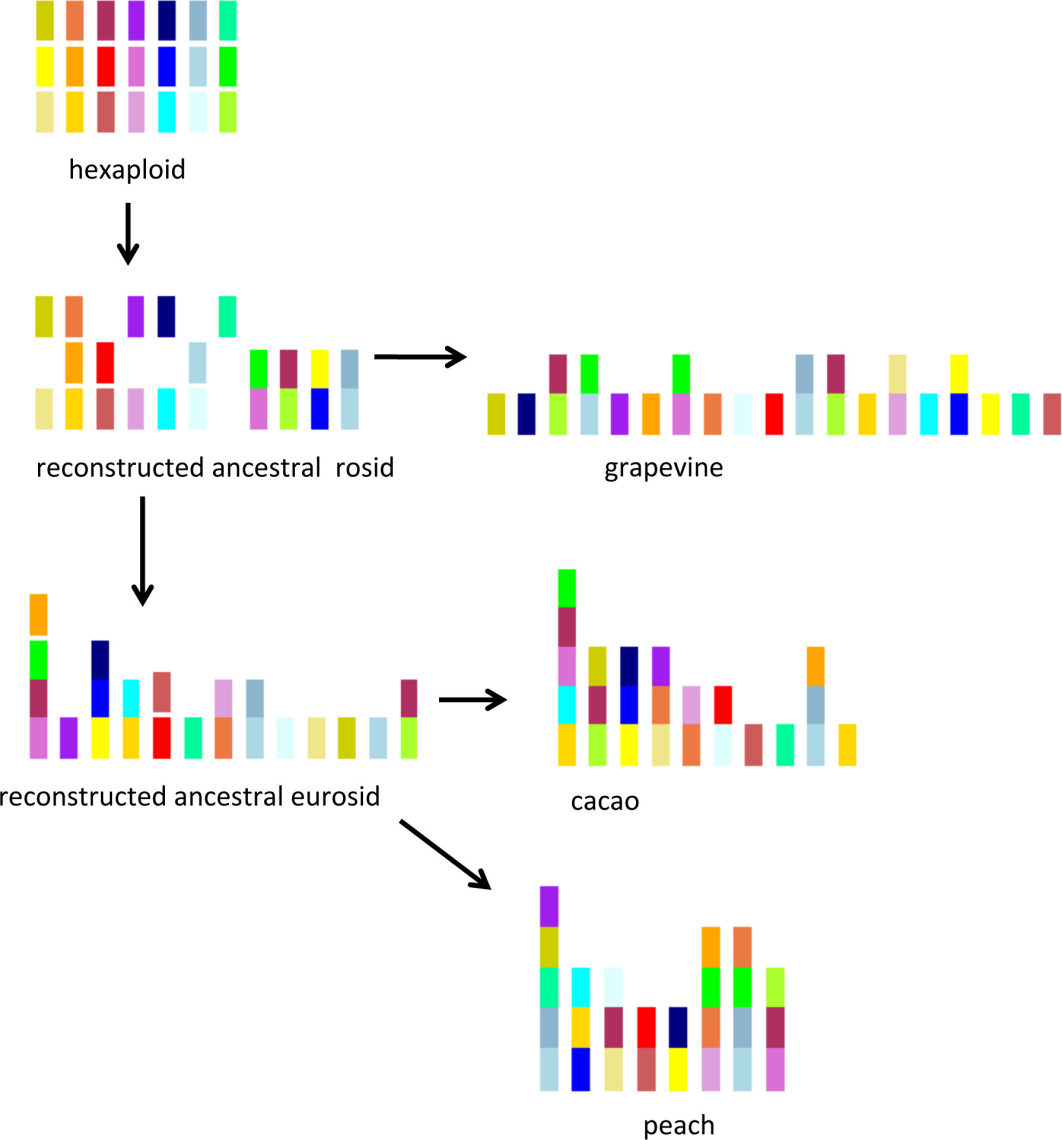
**From ancestors to modern genomes**. Schematic attribution of triplicated regions in the rosid and eurosid ancestors.

This analysis consists of the following hypotheses, observations and inferences, which are schematized on Figure 7.

1. The ancestral hexaploid is assumed to contain three identical sets of seven chromosomes, which we label with seven colors, each in three shades from darkest to lightest.

2. All three sequenced genomes have a large light green region adjacent to, or very close to, a small dark red region on the same chromosome, as well as another large dark red region elsewhere. This suggests a non-reciprocal translocation occurring on the tree branch between the hexaploid ancestor and the rosid ancestor, and accounts for one of the fused chromosomes depicted in the latter. There is also a small light green region at the end of the dark red chromosome in grapevine. This is most likely the result of a chromosomal fission in the tree branch leading to grapevine.

3. There are two chromosomes containing medium green in grapevine. This would have required a fission in the lineage leading to grapevine, but this must have been preceded by a fusion of medium green with medium purple in the common ancestor of grapevine and cacao, since this association is present in both. This accounts for another of the fused chromosomes apparent in the rosid ancestor.

4. All genomes show adjacencies between medium pale blue and dark pale blue, suggesting a fusion in the rosid ancestor as shown, with a further translocation with medium green in grapevine and additional rearrangements in peach.

5. Grapevine and cacao evidence an early fusion of medium blue and medium yellow (the remaining fused chromosome visible in the rosid ancestor), followed by a fission of most of the medium yellow to form a separate chromosome in grapevine.

6. There is an association of dark blue and medium yellow in the two eurosid genomes only, suggesting this event occurred after the divergence from Vitales. However, the inherited association with medium blue is retained in cacao, so that a three-way fusion is is portrayed in the eurosid ancestor. Other exclusively eurosid associations include fusions of light purple and dark orange, and of light blue and light orange, with much of the light orange fissioning to form a separate chromosome in cacao. In addition, the medium green/medium purple fusion inherited from the rosid ancestor has acquired an association with dark red in the eurosids, so that a three-way fusion seems appropriate there.

These considerations suggest, as summarized in Figure 7, that the rosid ancestor had 18 chromosomes, four resulting from fusions among the original hexaploid chromosomes, and 14 consisting of entire chromosomes or parts of single chromosomes from the latter. Grapevine would have undergone two additional fusions and two fissions (at new breakpoints) of chromosomes previously fused in the rosid ancestor.

Similarly the eurosid ancestor can be predicted to have at most 15 chromosomes, derived by four additional fusions and one fission, from which the 10-chromosome cacao and 8-chromosome peach evolved, mostly by further fusions.

## A comparison of gene-based and region-based reconstructions

Although the exercise in the previous section takes no account of the actual gene-order evidence, it does provide a plausibility check on the detailed reconstruction leading to Figure 6. The discrepancy between the 13-chromosome reconstruction in Figure 6 and the 15-chromosome version in Figure 7 can be accounted for in terms of the fusion of the light orange region with the medium green/medium purple/dark red chromosome in the detailed reconstruction and the fusion of two other red chromosomes. In addition, there is a translocation of the medium blue from its position in cacao (Figure 7) to its position in peach (Figure 6). The compositions of the four chromosomes affected in Figure 6 are otherwise unchanged, and the remaining nine are identical in the two approaches.

We can conclude that, with allowances for its superficial nature, the "top-bottom" derivation in the previous section gives results which accord well with the detailed reconstruction earlier in this paper. The sole problem is the position of the medium blue region, whose positions in the rosid genomes can only be explained by two coincidental fusions (in cacao and grapevine) or by an unlikely translocation of exactly this region, no more and no less, to another chromosome in peach. Of interest is that in the reconstruction of the 9-chromosome Rosaceae ancestor by Jung *et al. *[8] based on grapevine, peach and strawberry, two of reconstructed chromosomes have the same overall structure as peach 2 and 5; this does not resolve the quandary, but at least suggests that the evolutionary events involved predate the Rosaceae radiation.

## Conclusions

As a prelude to ancestral genome reconstruction, we have quantitatively documented the details of the triplicated regions in the seven rosid eudicots under study, and suggested a model for fractionation in two steps, one pre-radiation and the other post-radiation. Fitting the model to the data from seven rosids requires the assumption of an unrealistically low number of genes involved in the initial hexaploidization, suggesting a dependence of fractionation rate on gene class, an effect that is confirmed by differential functional associations of highly fractionated versus highly retained gene triples in grapevine, cacao and peach.

A major preoccupation of our reconstruction methodology was to avoid the non-uniqueness of optimal solutions, a problem endemic to one-step reconstructions based even partly on poorly supported adjacencies. The key to this is to discard all such adjacencies from the MWM solution to the median problem. We rely instead on a second application of the algorithm to summary genomes made up of projections back from the high-confidence contigs produced by the initial run.

Our reconstruction procedure requires three parameter settings. One is the weight system, which on one hand distinguishes between conflicting evidence and absent evidence against adjacencies with the same level of support, and on the other hand detects a certain number of poorly supported adjacencies whose contexts in the three genomes suggest that they nevertheless merit inclusion in a solution. The second parameter is a threshold for contig sizes (more than three adjacencies), designed to eliminate noise, due either to errors or insensitivity in the data-generation steps, or to movements of isolated genes to remote locations in the genome. Despite a general tendency to conserve the large triplicated regions, there is much low-level rearrangement occurring in these genomes. They each require from 400 to 600 rearrangements to be generated from the reconstructed ancestral gene order, though much of this is ascribable to fractionation; inevitably a few of these rearrangements conspire in two genomes to mislead the reconstruction, especially if we place too much faith in the significance of contigs containing only one or two genes. The third setting is the cutoff for merging neighboring fragments after the higher-level run of MWM. In our data, the appropriate value empirically obtained was 500 genes; 400 was too low, unable to detect several valid fusions, while 600 was too high, resulting in an unbalanced genome concentrated largely in a single chromosome. Of the three parameter settings, the first two should be generally applicable, while the third will have to be adjusted (in an automated way if desired, using chromosome number or other karyotype characteristic) to each data set. At least for our data set, all of these settings are indispensable. Not dropping small contigs after the first MWM leads to many unlikely fusions as well as numerous tiny chromosomes that do not fit in with the rest. Using simply weights 1, 2 and 3 instead of those in Table 3, or not using the right cutoff lead, both lead to unbalanced genomes and other anomalies.

As more angiosperm genome sequences become available, reconstruction of additional early ancestral genomes will become possible, adding to the Rosaceae ancestor in [8], and the eurosid ancestor studied here, to further elucidate the evolution of large clades of these plants. Additionally, genomes that break up the phylogenetic clades studied here will help resolve conflicting evidence of chromosome fusion and fission events. Paradoxically, although the whole genome doubling (or tripling) that recurs throughout this evolutionary domain has the effect of scrambling local gene order through fractionation, it also provides a convincing way of validating reconstructions based on gene order, as exemplified by the intact triplicated regions inferred in Figure 6.

This paper is based in part on an extended abstract published in the proceedings of the ICCABS 2012 conference [31]. The only sections that were retained, though extensively rewritten, are the first two sections after the Introduction, corresponding to Figures 2 - 4 in the present version. Virtually all of the rest of the original paper was discarded and replaced by new material.

## Competing interests

The authors declare that they have no competing interests.

## Authors' contributions

CZ and DS did the research and wrote the paper. EC carried out the functional analysis. VA and EL provided motivation, suggestions and interpretations throughout the project. All authors read and approved the manuscript.

## Declarations

Publication of this article was supported by the Canada Research Chair in Mathematical Genomics.

This article has been published as part of *BMC Genomics *Volume 14 Supplement 7, 2013: Selected articles from the Second IEEE International Conference on Computational Advances in Bio and Medical Sciences (ICCABS 2012): Genomics. The full contents of the supplement are available online at http://www.biomedcentral.com/bmcgenomics/supplements/14/S7
